# Integrated analysis of genes associated with poor prognosis of patients with colorectal cancer liver metastasis

**DOI:** 10.18632/oncotarget.16064

**Published:** 2017-03-10

**Authors:** Zhenyuan Qian, Guobing Zhang, Guangyuan Song, Ji Shi, Lijie Gong, Yiping Mou, Yong Han

**Affiliations:** ^1^ Department of Gastrointestinal Surgery, Zhejiang Provincial People’s Hospital, Hangzhou, Zhejiang 310014, P.R. China; ^2^ Department of Pharmacy, Zhejiang Provincial People’s Hospital, Hangzhou, Zhejiang 310014, P.R. China; ^3^ Zhejiang Chinese Medical University, Hangzhou, Zhejiang 310053, P.R. China; ^4^ Key Laboratory of Gastroenterology of Zhejiang Province, Zhejiang Provincial People’s Hospital, Hangzhou, Zhejiang 310014, P.R. China; ^5^ Clinical Research Institute, Zhejiang Provincial People’s Hospital, Hangzhou, Zhejiang 310014, P.R. China

**Keywords:** colorectal cancer, prognostic marker, liver metastasis

## Abstract

Colorectal cancer (CRC) is one of the most common malignances in the gut. Liver is the most common metastasis site of CRC. This study focuses on the primary CRC and its liver metastasis, aiming to discover several liver metastasis related genes and provide therapeutic candidates. We compared gene expression patterns among the groups of normal colorectal mucosa, primary tumor and the liver metastasis using a CRC gene expression dataset. 84 genes were found to be upregulated in both primary tumor and liver metastases. Function enrichment analysis indicated that these genes are enriched in pathways such as chemotaxis, coagulation and lipid metabolism which are crucial in multi-step cancer metastasis. Gene network analysis identified several important hub genes that may be involved in carcinogenesis and liver metastasis. Then we used a validation dataset containing 562 CRC samples with detailed clinical information, to screen prognostic biomarkers for overall survival (OS) and relapse free survival (RFS). Finally, overexpression of THBS2 (thrombospondin 2), INHBB (inhibin, beta B) and BGN (biglycan) were proved to be correlated with poor OS and RFS. In conclusion, this study indicated that chemotaxis, coagulation and lipid metabolism might play critical roles in the processes of carcinogenesis and liver metastasis. THBS2, INHBB and BGN are prognostic markers and potential therapeutic targets for CRC.

## INTRODUCTION

Colorectal cancer (CRC) is one of the most common malignances in the digest system [1–3]. Frequently, distal metastasis affects clinical outcomes. Colorectal cancer liver metastasis (CRCLM) is the most common metastasis pattern in CRC [4]. It is possible to have a radical surgery on advanced colorectal cancer accompanied with liver metastasis in certain cases [4, 5]. It is also probable to cure thoroughly by radical surgery, radio-chemotherapy, and molecular targeted therapy [6–9]. Unfortunately, such patients only accounts for a very small proportion while most CRCLM patients have poor outcomes. Therefore, two questions need to be addressed. Firstly, which subgroup of CRC patients will progress to liver metastasis? Secondly, which subgroup of patients with CRCLM will have poorer prognosis? Investigations on novel prognostic biomarkers and therapeutic targets are needed to improve the outcomes of CRCLM patients.

## RESULTS

### Flow chart of this study

**Figure 1:**
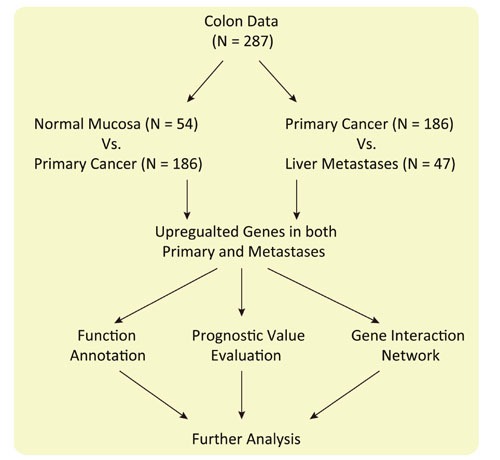
Flow chart of this study

This study focuses on the carcinogenesis and liver metastasis of CRC (Figure 1). Firstly, we searched Gene Expression Omnibus (GEO) for choosing appropriate dataset for further analysis (GSE68468). Then we compared the gene expression pattern between normal colon mucosa and primary tumors; primary tumor and liver metastasis, respectively. Genes upregulated in both primary tumors and liver metastasis were selected for function enrichment analysis, gene-gene interaction analysis and prognostic power evaluation.

### Identification of upregulated genes in both primary tumor and liver metastasis

**Figure 2:**
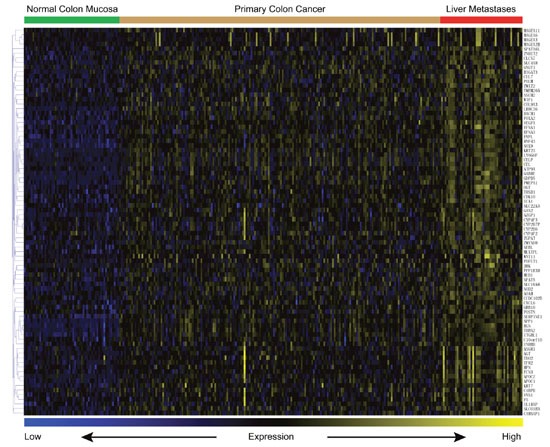
Heat map of 84 upregulated genes in both PT and LM

**Figure 3:**
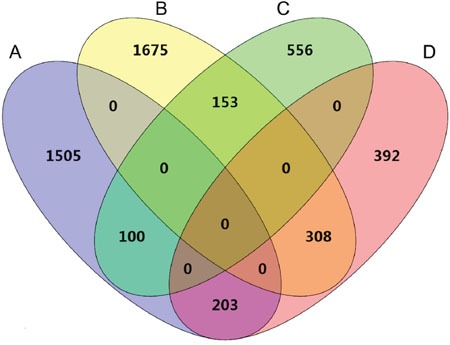
Venn diagram of differentially expressed genes in PT vs. N and PT vs. LM **(A)** genes upregulated in PT compared to N. **(B)** genes down regulated in PT compared to N. **(C)** genes upregulated in LM compared to PT. **(D)** genes down regulated in LM compared to PT.

**Figure 4:**
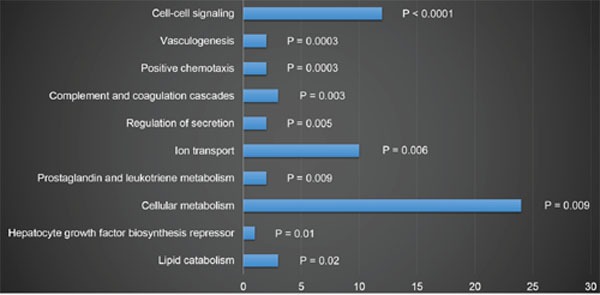
Function enrichment of 84 genes upregulated in both PT and LM

**Figure 5:**
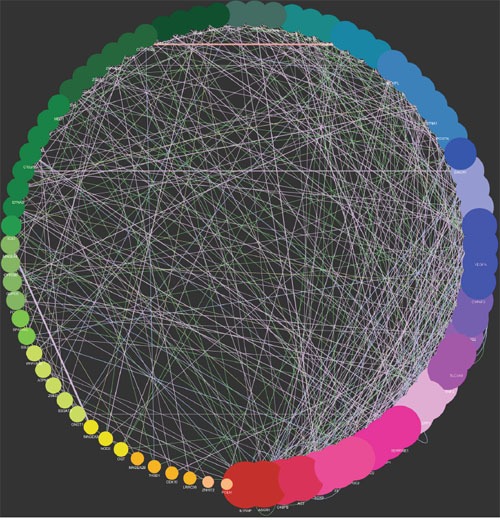
Gene-gene interaction network of 84 genes upregulated in both PT and LM

The genes expression patterns of 54 normal colon mucosa (N), 186 primary tumors (PT) and 54 liver metastases (LM) were compared to discover upregulated genes in both PT and LM. 100 probes representing 84 genes were found to be upregulated in both PT and LM (Figure 2 and Table 1). Heat map visualization of 84 upregulated genes in N, PT and LM samples was demonstrated in Figure 3. Function enrichment of these 84 upregulated genes indicated that chemotaxis, coagulation and lipid metabolism etc. were critical signaling pathways (Figure 4). Gene-gene interaction network was constructed (Figure 5) and hub genes were listed in Table 2 (degree ≥ 15).

**Table 1:** 84 genes upregulated in both primary tumor and liver metastasis

Gene Symbol	Gene Title
EFNA1	ephrin A1
SERPINE1	serpin family E member 1
AGT	angiotensinogen
SOX9	SRY-box 9
THBS2	thrombospondin 2
B3GAT3	beta-1,3-glucuronyltransferase 3
CDK10	cyclin dependent kinase 10
MED1	mediator complex subunit 1
CUL7	cullin 7
APOC1	apolipoprotein C1
EVPL	envoplakin
GABRE	gamma-aminobutyric acid type A receptor epsilon subunit
PPP1R3D	protein phosphatase 1 regulatory subunit 3D
APOC2	apolipoprotein C2
WIF1	WNT inhibitory factor 1
F5	coagulation factor V
COL9A3	collagen type IX alpha 3 chain
HPN	hepsin
INHBB	inhibin beta B subunit
SLC22A3	solute carrier family 22 member 3
ITGBL1	integrin subunit beta like 1
DACH1	dachshund family transcription factor 1
AOAH	acyloxyacyl hydrolase
VNN1	vanin 1
GAS2	growth arrest specific 2
FCN3	ficolin 3
CEL	carboxyl ester lipase
TDO2	tryptophan 2,3-dioxygenase
CXCL6	C-X-C motif chemokine ligand 6
SLCO1B3	solute carrier organic anion transporter family member 1B3
CYP4F2	cytochrome P450 family 4 subfamily F member 2
CYP4F3	cytochrome P450 family 4 subfamily F member 3
WNT11	Wnt family member 11
SSUH2	ssu-2 homolog (C. elegans)
ASGR1	asialoglycoprotein receptor 1
CYP2B7P	cytochrome P450 family 2 subfamily B member 7, pseudogene
CYP2B6	cytochrome P450 family 2 subfamily B member 6
SLC16A6	solute carrier family 16 member 6
SLC4A8	solute carrier family 4 member 8
ZMYND8	zinc finger MYND-type containing 8
GNGT1	G protein subunit gamma transducin 1
CELP	carboxyl ester lipase pseudogene
LY6G6F	lymphocyte antigen 6 complex, locus G6F
C4BPB	complement component 4 binding protein beta
KRT7	keratin 7
C10orf10	chromosome 10 open reading frame 10
CLCN7	chloride voltage-gated channel 7
AZGP1	alpha-2-glycoprotein 1, zinc-binding
SPP1	secreted phosphoprotein 1
MAGEA3	MAGE family member A3
FOXA2	forkhead box A2
EFNA3	ephrin A3
TFR2	transferrin receptor 2
IL1RAP	interleukin 1 receptor accessory protein
POFUT1	protein O-fucosyltransferase 1
MAGEA11	MAGE family member A11
VEGFA	vascular endothelial growth factor A
OGT	O-linked N-acetylglucosamine (GlcNAc) transferase
GDPD5	glycerophosphodiester phosphodiesterase domain containing 5
TMEM265	transmembrane protein 265
BGN	biglycan
ICA1	islet cell autoantigen 1
MAGEA2B	MAGE family member A2B
MAGEA6	MAGE family member A6
JRK	Jrk helix-turn-helix protein
POSTN	periostin
NFAT5	nuclear factor of activated T-cells 5
GRB10	growth factor receptor bound protein 10
ATP9A	ATPase phospholipid transporting 9A (putative)
NEBL	nebulette
PMEPA1	prostate transmembrane protein, androgen induced 1
RNF43	ring finger protein 43
KRT23	keratin 23
ZNHIT2	zinc finger HIT-type containing 2
THSD1	thrombospondin type 1 domain containing 1
LRRC36	leucine rich repeat containing 36
NOD2	nucleotide binding oligomerization domain containing 2
SPATA6L	spermatogenesis associated 6 like
CCDC102B	coiled-coil domain containing 102B
CAMSAP1	calmodulin regulated spectrin associated protein 1
MLXIPL	MLX interacting protein like
ZMIZ2	zinc finger MIZ-type containing 2
ZGPAT	zinc finger CCCH-type and G-patch domain containing
POLM	DNA polymerase mu

**Table 2:** Network analysis of 84 upregulated genes in both PT and LM (Degree≥15)

Gene Symbol	Degree	Average Shortest Path Length	Clustering Coefficient
ASGR1	28	1.74358974	0.29347826
IL1RAP	28	1.80769231	0.22529644
AGT	27	1.79487179	0.36758893
C4BPB	27	1.75641026	0.24675325
SOX9	25	1.80769231	0.21212121
APOC2	25	1.83333333	0.4
F5	25	1.82051282	0.34632035
TDO2	23	1.87179487	0.33986928
AZGP1	23	1.82051282	0.28063241
SERPINE1	23	1.80769231	0.27619048
INHBB	22	1.84615385	0.19883041
SPP1	22	1.88461538	0.24836601
CXCL6	22	1.87179487	0.27368421
SLC4A8	21	1.82051282	0.22222222
CYP4F2	21	1.91025641	0.19852941
HPN	20	1.82051282	0.31052632
SLCO1B3	19	1.88461538	0.35833333
THBS2	19	1.8974359	0.25735294
VEGFA	18	1.8974359	0.19852941
CYP4F3	18	1.97435897	0.32967033
APOC1	18	1.93589744	0.34065934
GAS2	18	1.92307692	0.2952381
TFR2	17	1.98717949	0.59090909
VNN1	17	1.8974359	0.34166667
KRT23	17	2	0.30769231
DACH1	16	1.94871795	0.175
FCN3	15	1.93589744	0.24761905
POSTN	15	2.02564103	0.23076923
EFNA1	15	1.97435897	0.37179487
COL9A3	15	1.93589744	0.28571429
BGN	15	1.93589744	0.26666667
MLXIPL	15	1.94871795	0.48351648

### Evaluation the prognostic power of 84 upregulated genes

**Figure 6:**
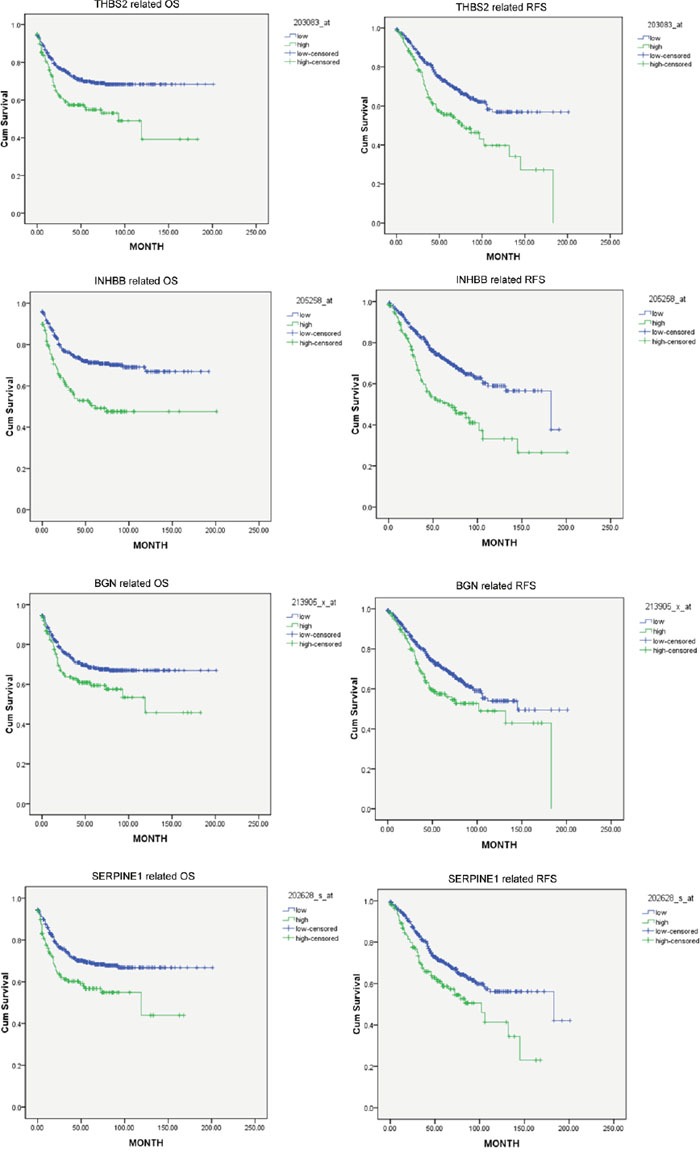
THBS2, INHBB, BGN, and SERPIN1 were correlated with poor OS and RFS Cut-off value was selected as the upper quartiles of each gene ranked by expression values.

A dataset containing 562 samples was utilized to evaluate the prognostic power of 84 upregulated genes. The expression of each gene was defined as high expression and low expression by quartile division at the cutoff of 25%, 50% and 75%. Then we analyzed the relationship between survival and gene expression at different cutoff level. Survival analysis results indicated that four hub genes, namely thrombospondin 2 (THBS2), inhibin beta B (INHBB), biglycan (BGN), and serpin peptidase inhibitor, clade E (nexin, plasminogen activator inhibitor type 1), member 1 (SERPIN1) were associated with both OS and RFS (Figure 6, cut-off value: upper quartiles of gene expression, p<0.05). Since the association between SERPIN1 and CRC metastasis has been reported [10, 11], we chose THBS2, INHBB and BGN for further study.

### The prognostic power of three hub genes in different tumor stages

**Figure 7:**
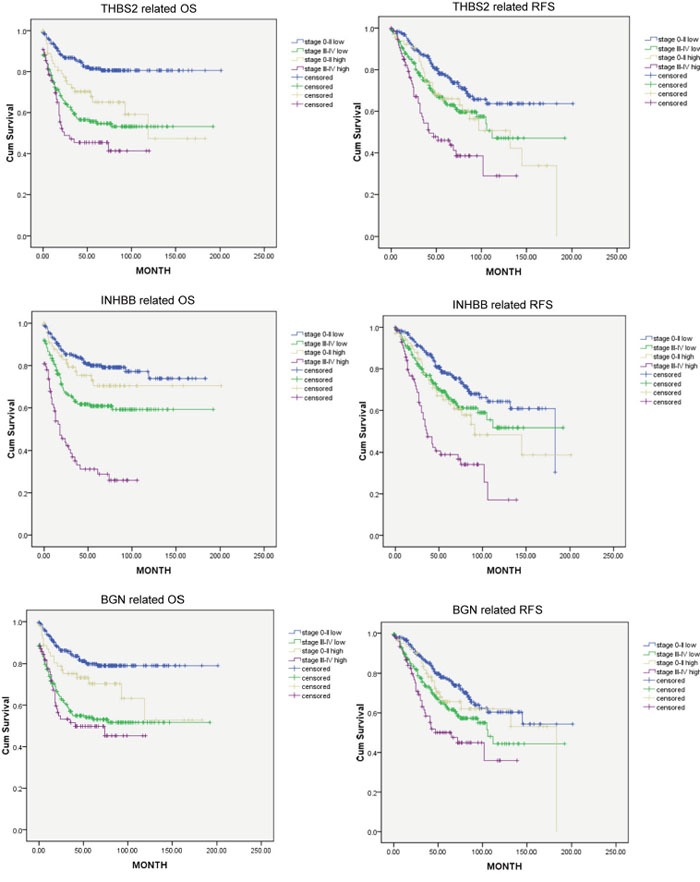
Kaplan-Meier plot analyses of THBS2, INHBB and BGN (cut-off value of high and low group: upper quartiles) in stage 0-II and stage III-IV

Patients were divided into groups of stage 0-II and stage III-IV. Then the prognostic power of each hub genes were tested on these two groups separately. Figure 7 indicated that prognostic power of THBS2 and INHBB were independent from tumor staging (cut-off value: upper quartiles, P<0.05). However, BGN was not an independent prognostic marker (cut-off value: upper quartiles, P>0.05).

### Combination of three hub genes for predicting OS and RFS

**Figure 8:**
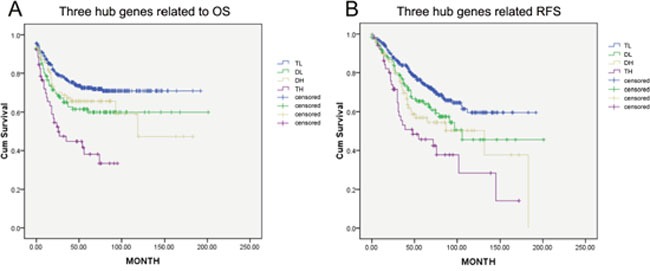
THBS2, INHBB and BGN were classified into four groups (TL, DL, DP and TH; cut-off value of high and low group: upper quartiles) OS and RFS were significantly different between these groups (p<0.05). TN has the best survival while TH the worst.

According to the expression level of three hub genes (cut-off value: upper quartiles), samples are divided into four groups: triple high expression, double high expression, double low expression and triple low expression, respectively (TH, DH, DL and TL). TL patients have the best survival for OS and RFS while the survival of TH patients are the worst (p<0.5). There was no significant difference between DL and DH groups (Figure 8).

## DISCUSSION

Colorectal cancer with liver metastasis (CRCLM) is the research hotspot in recent years [12]. As is known, liver is the most common metastasis site of colorectal cancer, because of dissemination via the portal venous system. About 10% CRCLM can be resected by surgery, however, only 25%-50% of them could be free of recurrence after treatments [13]. Cheng D et al. reported that MicroRNA -20a-5p, as an intermediator, may promote the ability of invasion and metastasis in colorectal cancer by suppressing Smad4 [14]. Mismatch repair deficiency status, such as frequent PIK3CA mutation, was also seemed as a cause to carcinogenesis in colorectal cancer [15, 16]. Moreover, microsatellite instability in colorectal cancer was also very important. For example, methylation of ITF2 plays a role in modulating WNT signaling in CRC [17]. However, key gene regulators related to carcinogenesis and metastasis of CRC are still unclear. Therefore, discovering novel genes associated with CRCLM and distinguishing new prognostic biomarkers are critical for managing patients with CRCLM.

In this study, 84 genes were found to be associated with the carcinogenesis and liver metastasis of CRC. Chemotaxis, coagulation and lipid metabolism are key signaling pathways involved in carcinogenesis and liver metastasis. By performed gene-gene interaction network analysis, 32 hub genes were discovered. Kaplan-Meier analysis was performed for each gene separately, and SERPINE1, THBS2, INHBB and BGN were found to be associated with poorer OS and RFS.

Metastasis to distant organs is a multistage process: local invasion, intravasation, survival in the circulation, extravasation and colonization [18]. The activation of the coagulation system and platelets have a critical role in the progression of cancer [19]. By forming cell aggregates that protect tumour cells from immune surveillance or by collaborating during extravasation, tumour cell-platelet interactions could also enable dissemination [20–23]. Chemotaxis and vasculogenesis in cancer are crucial for increasing vascular permeability which could improve extravasation and lead to metastasis eventually [19]. In the present study, we showed that chemotaxis and coagulation processes etc. are key characteristics CRC progression and are potential therapeutic targets.

SERPINE1 encodes a member of the serine proteinase inhibitor superfamily which is the principal inhibitor of tissue plasminogen activator (tPA) and urokinase (uPA), and hence is a inhibitor of fibrinolysis. It reacts directly to integrin, uPA-uPAR and ECM, causing to invade and migrate to surrounding and distance [10, 24]. Meanwhile, SERPINE1 has been considered as biomarker related to poorer prognosis in CRC [11]. Since there are few reports on THBS2, INHBB and BGN in CRC, especially in CRCLM, we chose these three genes for further analysis.

THBS2, a member of thrombospondin family, is a disulfide-linked homotrimeric glycoprotein that mediates cell-to-cell and cell-to-matrix interactions, and hence modulates the cell adhesion and migration. Initially, THBS2 was thought to be related to heart failure [25]. Subsequently, it’s also found linked to poorer prognosis in oral cavity squamous cell carcinoma [26]. Interestingly, in many other carcinomas, THBS2 was found downregulated and was correlated with poorer prognosis in tumors such as gastric cancer [27, 28], prostate cancer [29] and breast cancer [30]. In the present study, we found that THBS2 was overexpressed in both primary colon tumor and liver metastases. Moreover, its expression was negatively correlated with OS and RFS.

INHBB is the subunit of inhibin, which regulates gonadal stromal cell proliferation negatively and has tumor-suppressor activity. It seems as an independent prognostic parameter in uterine non-endometrioid cancer, and a low expression demonstrates a significant better cause-specific survival [31]. Investigating the effects of INHBB gene knockdown on the development of mouse granulosa cells *in vitro*, Mohamed et al. found INHBB has the function of inhibiting apoptosis in mouse granulosa cell [32]. However, the role of INHBB in gastrointestinal cancer, especially in CRC, has not been thoroughly studied from now on. Our analysis confirmed the overexpression of INHBB in CRCLM and its association with poorer OS and RFS, which may result from its function of inhibiting apoptosis.

BGN gene encodes a member of the small leucine-rich proteglycan family of proteins. The encoded protein may contribute to atherosclerosis and aortic valve stenosis in human patients. It seems to enhance the ability of migration and invasion in endometrial cancer [33]. BGN also promotes tumor invasion and metastasis of gastric cancer both *in vitro* and *in vivo* [34]. Activated FAK signaling pathway, which regulates cell adhesion and motility by relaying ECM signals from integrin to the intracellular compartment, leads to tumor invasion and metastasis [35]. However, there are few reports on BGN in CRC. In the present study, BGN was found overexpression in CRCLM, and it was correlated with poorer OS and RFS. The underlying molecular mechanism was not clear at present, but FAK signaling pathway may take an important role in this process.

The correlation among three hub genes and survival were also explored in respective to TNM stage. THBS2 and INHBB were independent prognostic biomarker for OS and RFS in both stage 0-II and stage III-IV, while the prognostic value of BGN was associated with TNM staging. Nevertheless, synchronous high expression of these three hub genes indicates the worst clinical outcome, while synchronous low level indicates the best, emphasizing their contributions to poor prognosis.

In summary, several critical signaling pathways such as chemotaxis, coagulation and lipid metabolism may have critical roles in the processes of CRC carcinogenesis and liver metastasis. THBS2, INHBB and BGN are prognostic markers and potential therapeutic targets for CRC. Validation of large cohorts and wet lab experiments are still needed before achieving any clinical significance.

## MATERIALS AND METHODS

### Ethics statement

The databases used in our study are available online. Anyone is permitted to use all the data in the website of ArrayExpress (http://www.ebi.ac.uk/arrayexpress/), which contains more than 65 thousands of experiments and about 2 millions of assays. It also supports for a search facility to get what type of array and the related information we want. Gene expression datasets were downloaded from Gene Expression Omnibus (GEO) website (www.ncbi.nlm.nih.gov/geo/), which can be linked in the experiments searched out in ArrayExpress. GEO is a public functional genomics data and open for everyone. It contains more than 4300 datasets, 77000 series and 2000000 samples. It provides us some useful tools, such as GEO2R, to get the information we needs. Since all the data are publicly available, The Research Ethics Committee of Zhejiang Provincial People’s Hospital therefore waived the requirement for ethical approval.

### Selection of databases

We searched in the ArrayExpress with the condition of colorectal adenocarcinoma and liver metastasis, filtered by organism of Homo sapiens, experiment type of array assay and RNA assay. As a result, we searched out 29 experiments that satisfied the conditions. According to the total sample capacity and the volume of colorectal mucosa, primary tumor and liver metastasis respectively, we chose GSE68468 as the database looking for upregulated genes both in primary CRC and in liver metastasis. Then we re-searched the ArrayExpress with the condition of colorectal adenocarcinoma, filtered by organism of Homo sapiens, experiment type of array assay and RNA assay. As a result, there are 479 experiments satisfied. According to the data-integrity and sample quantity, we focused on the dataset of GSE40967 on the platform of GPL570, which contains the follow-up of OS and RFS in 562 samples testing the gene expression of primary tumor.

### Genomic analyses

GEO dataset [36] GSE68468 [37], which containing 54 normal colon mucosa, 186 primary tumors and 47 liver metastases, was reanalyzed for discovering gene associated with both colon carcinogenesis and liver metastasis. While GSE40967 [38] was used for validating the prognostic value of these genes in 562 patients with colon cancer. Differentially expressed genes were computed using Limma package in R environment (version 3.2.1, R Foundation for Statistical Computing [http://www.r-project.org/]). Venn diagram was plotted by Venny online software (Oliveros, J.C. [http://bioinfogp.cnb.csic.es/tools/venny/index.html]). MeV was employed for Heat map and clustering analyses [39]. Function enrichment was performed using GATHER [40], while gene-gene interaction network was constructed by utilizing GeneMANIA plugin [41].

### Statistical analyses

Differentially expressed genes were selected by the following criteria: P < 0.05, Log 2 Transformed Fold Change > 0.6 or < -0.6. Kaplan-meier plot analysis was performed using SPSS software (IBM, version 21.0) and log-rank test was employed to evaluate statistical significance. All the data were analyzed using standard statistical tests. Significance was defined as P value less than 0.05.
